# Health related quality of life in patients in dialysis after renal graft loss and effect of gender

**DOI:** 10.1186/1472-6874-14-34

**Published:** 2014-03-01

**Authors:** Nanna von der Lippe, Bård Waldum, Tone-Brit Hortemo Østhus, Anna Varberg Reisæter, Ingrid Os

**Affiliations:** 1 Institute of Clinical Medicine, University of Oslo, Oslo, Norway; 2 Department of Nephrology, Ullevål, Oslo University Hospital, Oslo, Norway; 3 Department of Transplantation Medicine, Rikshospitalet, Oslo University Hospital, Oslo, Norway

**Keywords:** Dialysis, Kidney transplant failure, HRQOL, Gender

## Abstract

**Background:**

An increasing number of dialysis patients have returned to dialysis after renal graft loss, and the transition in disease state could likely be associated with reduced health related quality of life (HRQOL). Furthermore, gender differences in HRQOL have been observed in dialysis and kidney transplanted patients, but whether transition in disease state affects HRQOL differently in respect to gender is not known. The aims of this study were to compare HRQOL in dialysis patients with graft loss to transplant naïve dialysis patients, and to explore possible gender differences.

**Methods:**

In a cross-sectional study, HRQOL was measured in 301 prevalent dialysis patients using the Kidney Disease and Quality of Life Short Form version 1.3. Adjusted comparisons were made between dialysis patients with previous graft loss and the transplant naïve patients. Multiple linear regression analyses were performed with HRQOL as outcome variables. Interaction analyses using product terms were performed between gender and graft loss. HRQOL was analysed separately in both genders.

**Results:**

Patients with renal graft loss (n = 50) did not experience lower HRQOL than transplant naïve patients after multiple adjustments. Among patients with graft loss, women (n = 23) reported lower HRQOL than men (n = 27) in the items physical function (40 vs. 80, p = 0.006), and effect of kidney disease (49 vs. 67, p = 0.017). Women with graft loss reported impaired kidney-specific HRQOL compared to transplant naïve women (n = 79) in the items effect of kidney disease (50 vs. 72, p = 0.002) and cognitive function (80 vs. 93, p = 0.006), and this observation persisted after multiple adjustments. Such differences were not apparent in the male counterparts.

**Conclusions:**

Patients who resumed dialysis after renal graft loss did not have lower HRQOL than dialysis patients not previously transplanted. However, losing graft function was associated with reduced HRQOL in females, and important interactions were identified between graft loss and gender. This needs to be further explored in prospective studies.

## Background

It is imperative to improve health related quality of life (HRQOL) in dialysis patients, as this repeatedly has been shown to be low compared to the general population as well as to patients with other chronic diseases [1,2]. Renal transplantation is considered the optimal renal replacement therapy, and convergent research has shown that transplantation improves HRQOL compared to dialysis [3,4]. Between 15-25% of kidney transplanted patients experience graft loss during the first five years [5,6], and this may be perceived as a great health threat to the patients [7]. An increasing number of transplanted patients with graft loss will recommence dialysis [8].

There is a scarcity of data on HRQOL in dialysis patients with functional graft loss, and the few and small studies report divergent results [9-12]. Results from Dialysis Outcomes and Practice Pattern Study (DOPPS) were recently published, adding important knowledge about outcome in dialysis patients after transplant failure [13]. Intuitively and based on clinical experience, we would expect patients with loss of renal graft function to perceive impaired HRQOL compared to other dialysis patients. Renal transplantation has been claimed to provide the greatest benefit regarding HRQOL [10,14], and the transition in the disease state to dialysis, would likely be associated with reduced HRQOL. Immunosuppressive therapy is usually maintained in lowered doses in Norway after graft loss, and this may affect the HRQOL [15].

Men are more likely to develop chronic renal disease [16], and they comprise a higher percentage of the incident and prevalent population of ESRD in most countries [17,18]. In the general population, HRQOL is perceived lower in women than in men [19], and this observation has been extended to renal transplant patients [10,14], while the results from dialysis patients are less consistent [20,21]. To our knowledge, studies have not previously addressed gender aspects of HRQOL in dialysis patients with previous graft loss.

Thus, the aims of this study were twofold: first, to explore the hypothesis that patients resuming dialysis after functional graft loss have impaired HRQOL compared to dialysis patients not previously transplanted; second, to assess whether there are gender differences in the two groups.

## Methods

### Study patients and design

**Figure 1:**
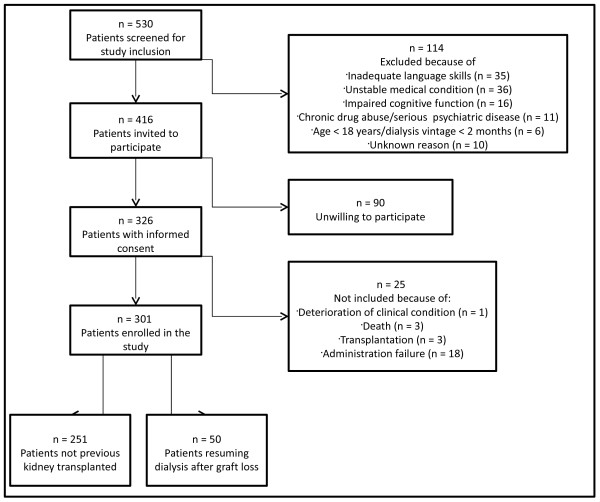
Flowchart of recruitment process.

Recruitment procedure and study design have been described previously [21]. All adult patients (≥18 years) receiving hemodialysis (HD) or peritoneal dialysis (PD) were screened for study participation at 10 different dialysis units in Norway, and could be included if they had received dialysis for more than 2 months. Patients were recruited from August 2005 to February 2007. A total of 301 prevalent dialysis patients participated in the cross-sectional study. Fifty dialysis patients had previously undergone renal transplantation (RG +), while 251 dialysis patients had not previously been transplanted (RG -) (Figure 1). If patients were hospitalized, they could not be included in the study, but could be enrolled 4 weeks or more after hospital discharge if they were in stable clinical condition. The study required adequate Norwegian language skills. Oral and written information were provided to the patients, and signed informed consent was required for enrolment. Cognitive dysfunction, psychosis or drug abuse were exclusion criteria. Enrolment rate was 72.4% [21]. Self-administered questionnaires were answered in a standardized fashion during treatment sessions for HD patients or during a scheduled visit for PD patients. Study nurses and physicians had been trained in applying the questionnaires which were distributed to the patients [21]. The Regional Committees for Medical and Health Research Ethics in Norway approved the protocol, and concession was obtained from the National Data Inspectorate. The study was accomplished according to the Helsinki Declaration [22].

### Demographic and clinical data

Demographic and clinical data were collected from hospital charts and/or direct questioning of the patients. Causes of renal failure, dialysis modality, dialysis vintage, comorbidities, history of previous transplant failure, and laboratory values were ascertained from medical records. Records of renal transplantation were based on the Norwegian Renal Registry [23]. Comorbidity was measured using the modified Charlson comorbidity index (CCI) [24]. CCI is validated for dialysis patients [24] and kidney transplanted patients [25], and has been shown to be a strong predictor of clinical outcomes [24,25]. CCI is a composite score of age and 17 weighted comorbid conditions including amongst others coronary artery disease, congestive heart failure, cerebrovascular disease, diabetes, malignancy and chronic pulmonary disease. In this study, CCI was calculated without including age in order to enable evaluation of age as a separate variable in multivariate analysis. Missing data were treated by pairwise deletion in the statistical analyses.

### Assessment of HRQOL

The Kidney Disease and Quality of Life Short Form version 1.3 (KDQOL-SF) [26] was applied to assess generic and kidney disease-specific HRQOL. The kidney disease-specific portion of KDQOL-SF consists of 43 questions classified into 11 specific kidney-related items: symptoms, effect of kidney disease, burden of kidney disease, work status, cognitive function, quality of social interaction, sexual function, sleep, social support, dialysis staff encouragement, and patient satisfaction. The second part comprises the widely used Medical Outcome Study 36-item Short Form Health Survey (SF-36) [27], which consists of 36 questions measuring the generic dimensions of HRQOL, grouped into eight items: physical function, role limitation due to physical problems, bodily pain, general health, vitality, social function, role limitation due to emotional problems, and mental health. All scores in KDQOL were transformed into linear 0- to 100 point scores, with higher score signifying a more favorable perception. Physical (PCS) and mental component summary (MCS) scores were calculated based on these items [28].

### Statistical analyses

Descriptive data were presented as either mean ± standard deviation (SD), or median with interquartile range (IQR) if data were skewed. Proportions were given for categorical variables. For comparisons between two groups, Student *t*-test was used for normal distributed data, and the Mann-Whitney test was used for skewed data. Chi-square test was used to compare categorical variables. Inequalities in the different items of HRQOL between RG + and RG – were checked. If the items differed, multiple linear regressions were performed with the HRQOL item as outcome variable. The SF-36 item physical function was also included as an outcome variable, as this item has been shown to be reduced in both dialysis patients and kidney transplanted patients [3,20,21]. To identify explanatory variables to be used in the different regression models, correlation coefficients (Spearman rho) between the HRQOL item “effect of kidney disease” and demographic and clinical variables were calculated. Variables with p < 0.1 could be entered in the regression models together with age, gender and graft loss. Preliminary analyses were conducted to ensure that the assumptions of normality, linearity, multicollinearity and homoscedasticity were not violated. Time in dialysis and body mass index (BMI) did not fulfill the assumptions of linearity, and were dichotomized with cut-off at the median time in dialysis (10 months), and BMI with the standardized cut-off point for overweight, (BMI ≥ 25 kg/m^2^).

The product term of gender and graft loss was entered into the multivariate regression models to check for gender interaction. Identification of such interaction would lead to the necessity of gender-specific multivariate analyses. All data were analysed using SPSS for Windows version 21 (IBM SPSS Statistics, New York, USA). A p level < 0.05 was considered significant.

## Results

### Clinical and demographic characteristics

There were low numbers of missing data, for SF-36 2% overall, and for KDQOL < 3% except the items effect of kidney disease (11%), dialysis staff encouragement (17%), satisfaction with care (16%) and sexual function (45%). Clinical and demographic characteristics of the study patients are given in Table 1. Differences between patients not previously transplanted (RG -) and patients resuming dialysis after graft loss (RG +) were observed for age, BMI and serum cholesterol. Additionally, a higher proportion was women and fewer patients had nephrosclerosis as cause of renal failure in the RG + group compared to RG - group. All RG + patients were still on immunosuppressive regimen in lowered doses. Half of the patients in the RG + group were on the waiting list for transplantation; this number did not differ from the RG – patients (Table 1). In the subgroup of females, patients in the RG + group were significantly younger than in the RG – group, 50.2 ± 14.9 vs 60.9 ± 15.7 years, respectively, p = 0.004.

**Table 1:** Patient characteristics of prevalent dialysis patients

	**RG + (n = 50)**	**RG – (n = 251)**	**p level**
**Female gender**	23/50	79/250	0.05
**Age years**	50.3 (16.6)	61.7 (15.5)	< 0.001
**Systolic blood pressure mmHg**	137 (23)	142 (21)	0.12
**Diastolic blood pressure mmHg**	79 (15)	77 (12)	0.46
**Body mass index kg/m**^**2**^	21.5 (20 - 25)	24.8 (22 - 28)	< 0.001
**Hemoglobin g/dL**	12.2 (1.5)	12.1 (1.5)	0.66
**Albumin g/L**	37.4 (4.7)	38.4 (5.0)	0.19
**CRP mmol/L**	8 (2 - 13)	6.0 (2 - 13)	0.92
**Cholesterol mmol/L**	4.7 (1.3)	4.2 (1.3)	0.01
**PTH pmol/L**	25 (12 - 51)	24 (13 - 40)	0.61
**Urea mmol/L**	23 (7)	22 (6)	0.18
Charlson comorbidity index^*^	3 (2 - 4)	4 (2 - 5)	0.08
**Dialysis vintage months**	13 (5 - 28)^† ^	10 (5 - 23)	0.52
**Nephrosclerosis**	3/48	76/248	< 0.001
**Waiting listed**	25/50	97/250	0.14

RG + = Dialysis patients with renal graft loss; RG - = Dialysis patients not previously transplanted. Data given as mean ± SD (standard deviation), median with IQR (inter quartile range), or proportion.

*Modified Charlson comorbidity Index without adding points for each decade of increasing age > 40 years; ^† ^Months after graft loss.

### Effect of gender and graft loss on HRQOL

**Figure 2:**
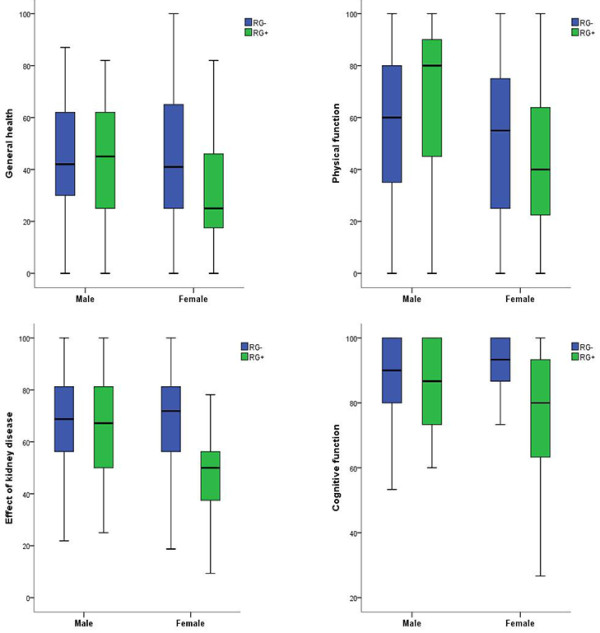
Box plots of subscales of SF-36* (upper panel) and KDQOL^**† **^**(lower panel) in RG - **^**‡ **^**and RG +**^**§**^**patients. **RG - (blue bars) and RG + (green bars). Significant differences between women and men with graft loss appeared in effect of kidney disease, p = 0.017 and physical function, p = 0.006. Differences between women with and without graft loss appeared in effect of kidney disease, p = 0.002 and cognitive function, p = 0.006. Non-significant results are not listed. *The Short Form Health Survey, ^† ^KDQOL = Kidney Disease and Quality of Life, ^‡^RG - = Dialysis, not previously transplanted, n = 251, ^§^RG + = Patients in dialysis with renal graft loss n = 50. Scores 0 -100, higher number indicating better QOL.

In unadjusted analyses, the kidney-specific items effect of kidney disease and cognitive function were perceived poorer in the RG + than in the RG - group (Table 2), while the generic item general health tended to be reduced in the RG + group (p = 0.06). No differences were observed in the other HRQOL items (Table 2). In the multivariate regression model for the total cohort, graft loss was not associated with kidney-specific or generic HRQOL. Female gender was associated with poorer self-perceived physical function than in males (score difference after multiple adjustment -7.6, p = 0.02). Higher age was associated with better scores in effect of kidney disease (0.4/year, p <0.001), general health (0.2/year, p = 0.01) and cognitive function (0.2/year, p = 0.001). Age was associated with lower scores in physical function (-0.5/year, p <0.001). Increased comorbidity was independently associated with lower scores in effect of kidney disease (-3.0/1 point CCI, p <0.001), worse general health (-3.9/1 point CCI, p < 0.001) and worse physical function (-4.5/1 point CCI, p < 0.001).No differences in HRQOL appeared between transplant-naïve and previous transplanted male dialysis patients, while women with previous graft loss had significantly lower scores in the unadjusted analyses compared to the transplant-naïve women (Figure 2). In the group with previous graft loss (RG+), women reported significantly lower HRQOL scores compared to the male counterpart (Figure 2), but no gender difference appeared in the group of transplant-naïve patients.

**Table 2:** KDQOL-SF 36 scores in prevalent dialysis patients

**KDQOL-SF36**	**RG + (n = 50)**	**RG – (n = 251)**	**p value**
***KDQOL:***			
**Symptoms**	71 (25 - 100)	75 (63 - 85)	0.24
**Effect of kidney disease**	56 (44 - 78)	69 (56 - 81)	0.01
**Burden of kidney disease**	31 (19 - 51)	31 (19 - 50)	0.84
**Work status**	0 (0 - 0)	0 (0 - 38)	0.43
**Cognitive function**	87 (67 - 93)	93 (80 - 100)	0.04
**Quality of social interaction**	90 (73 - 100)	87 (67 - 100)	0.49
**Sexual functioning**	63 (25 - 100)	62 (31 - 94)	0.99
**Sleep**	60 (46 - 78)	65 (48 - 80)	0.41
**Social support**	83 (67 - 100)	83 (67 - 100)	0.43
**Staff encouragement**	88 (75 - 100)	88 (75 - 100)	0.71
**Patient satisfaction with care**	83 (67 - 100)	83 (67 - 100)	0.26
***SF 36:***			
**Physical function**	55 (30 - 83)	58 (30 - 78)	0.65
**Physical role limitations**	0 (0 - 50)	0 (0 - 50)	0.56
**Bodily pain**	47 (32 - 77)	52 (41 - 84)	0.23
**General health**	35 (20 - 55)	42 (30 - 62)	0.06
**Vitality**	45 (24 - 55)	45 (30 - 60)	0.39
**Social function**	69 (38 - 88)	75 (50 - 88)	0.64
**Emotional role limitations**	33(0 - 100)	67 (0 - 100)	0.98
**Mental health**	72 (60 - 88)	80 (64 - 88)	0.30
PCS*	37 (± 12)	37 (± 19)	0.95
**MCS**^**† **^	46 (± 11)	48 (± 11)	0.44

RG + = Dialysis patients with renal graft loss; RG - = Dialysis patients not previously transplanted.

KDQOL = Kidney Disease and Quality of Life. SF-36 = The Short Form Health Survey. Data given as median with IQR (inter quartile range). Scores from 0-100, higher score signifying better quality of life.

*Physical component summary scale; ^**† **^Mental component summary scale. PCS and MCS are given as mean ± SD (standard deviation).

As significant interactions appeared between gender and graft loss for effect of kidney disease (p = 0.013), physical function (p = 0.028), and cognitive function (p = 0.017) in the multiple regression models, repeated multiple regression analyses were undertaken for men and women separately (Table 3). These analyses revealed that graft loss was associated with lower scores in effect of kidney disease, poorer physical function and worse self-perceived cognitive function and general health in women only (Table 3).

**Table 3:** Associations of explanatory variables on dimensions of HRQOL in female and male dialysis patients

**FEMALE GENDER (n = 90)**	**Effect kidney disease**			**Physical function**			**Cognitive function**			**General health**		
	**R**^**2**^**= 0.29, p <0.001**			**R**^**2 **^**= 0.27, p < 0.001**			**R**^**2 **^**= 0.15, p = 0.03**			**R**^**2 **^**= 0.18, p = 0.009**		
	**β**	**t**	**p**	**β**	**t**	**p**	**β**	**t**	**p**	**β**	**t**	**p**
Graft loss yes/no	-14.55	-2.49	0.015	-17.8	-2.59	0.01	-11.65	-2.41	0.018	-12.38	-2.04	0.045
Age +1 year	0.42	2.70	0.009	-0.50	-2.69	0.009	0.20	1.55	0.13	0.21	1.31	0.20
Comorbidity* +1 point	-4.75	-3.34	0.001	-5.85	-3.51	0.001	-1.22	-1.03	0.31	-4.28	-2.89	0.005
BMI^† ^≥ 25.0 kg/m^2^ yes/no	6.69	1.42	0.16	-0.45	-0.08	0.94	5.43	1.40	0.17	7.76	1.59	0.12
Dialysis vintage^‡^ ≥ 10 months yes/no	-2.56	-0.52	0.61	0.54	-0.09	0.93	0.35	0.09	0.93	3.91	0.77	0.45
Nephrosclerosis yes/no	-8.83	-1.40	0.17	-10.66	-1.44	0.15	-9.39	-1.79	0.08	-12.50	-1.90	0.06
**MALE GENDER (n = 197)**	**Effect kidney disease**			**Physical function**			**Cognitive function**			**General health**		
	**R**^**2 **^**= 0.17, p < 0.001**			**R**^**2 **^**= 0.23 p < 0.001**			**R**^**2 **^**= 0.06 p = 0.11**			**R**^**2 **^**= 0.16, p<0.001**		
	**β**	**t**	**p**	**β**	**t**	**p**	**β**	**t**	**p**	**β**	**t**	**p**
Graft loss yes/no	2.33	0.59	0.56	3.04	0.55	0.58	2.63	0.70	0.49	-1.67	-0.37	0.71
Age +1 year	0.38	4.28	<0.001	-0.49	-3.98	<0.001	0.25	2.93	0.004	0.24	2.42	0.016
Comorbidity* +1 point	-2.12	-2.74	0.007	-4.03	-3.74	<0.001	0.34	0.46	0.65	-4.04	-4.63	>0.001
BMI^† ^≥ 25.0 kg/m^2^ yes/no	7.46	2.84	0.005	-0.08	-0.02	0.98	1.49	0.60	0.55	3.62	1.22	0.23
Dialysis vintage^‡^ ≥ 10 months yes/no	-5.83	-2.19	0.03	-8.52	-2.30	0.02	-1.88	-0.75	0.46	-9.08	-3.03	0.003
Nephrosclerosis yes/no	-1.24	-0.42	0.66	3.32	0.81	0.42	-3.20	-1.15	0.25	-1.45	-0.44	0.66

Multiple regression analyses. Scores 0 -100, higher number indicating better QOL.

*Charlson comorbidity index without age; ^† ^Body mass index, dichotomized above or below 25.0 kg/m^2^.

^‡^Time in dialysis dichotomized with above or below the median value of 10 months.

Albumin, CRP, systolic blood pressure and cholesterol were not significant in univariate analyses, and therefore not included.

In the whole study group, men reported lower scores on sexual function compared to women, median scores 63 (25 - 88) vs. 75 (50 - 100) respectively, p = 0.006. The response rate was low for that particular question (56% for males vs. 54% for females).

A higher proportion of males than females had nephrosclerosis in the RG - group, 34.7 vs. 22% respectively, 59/170 vs. 17/78, (Χ^2^ = 4.19, p = 0.041).

## Discussion

As no difference in the adjusted HRQOL could be found between patients resuming dialysis after functional graft loss and dialysis patients who had never been transplanted, our hypothesis that patients recommencing dialysis after functional graft loss have impaired HRQOL compared to other dialysis patients, had to be rejected. The present study is, as far as we know, one of the largest comparing HRQOL in patients resuming dialysis after graft loss to other dialysis patients. Our data support the findings in a very small cross-sectional study with only nine patients with transplant failure, but no adjustment for other variables was done in that analysis [11]. In a longitudinal study, HRQOL was measured in 28 patients resuming dialysis after graft loss. The transition in treatment status from transplanted to dialysis patient was associated with reduced HRQOL and more symptoms and illness disruptiveness [12].

During the writing of this manuscript, DOPPS (Dialysis Outcomes and Practice Patterns Study) reported increased mortality in transplant failure patients compared to transplant naïve patients wait-listed for transplantation [13]. Secondary aims in DOPPS included effect of transplant failure on HRQOL. Physical component summary scale, but not mental component summary scale, was lower in the transplant failure patients. In our study, physical and mental component scores did not differ significantly between the patients with graft loss and the patients not previously transplanted. A similar proportion of transplant naïve and transplant failure patients were on the waiting list for transplantation in the present study, while in the DOPPS, only waiting listed patients in the transplant naïve group were included. The proportion of waiting listed patients in the transplant failure patients was substantial lower (25%). This could at least in part explain the divergent results in HRQOL between DOPPS and the present study. Actually, PCS and MCS scores in the previously transplanted patients in the two studies were quite similar, and the divergent conclusions might be explained by differences in the comparator groups. We have previously reported that dialysis patients on the waiting list for renal transplantation are doing better than those rejected for future transplantation [29]. The prospect of getting a retransplantation is high in Norway, the median time on the waiting list overall is 7 months [23], and this may have attenuated presumed differences between the two groups of dialysis patients in the present study.

A novel observation in the present study was the gender differences that were observed in HRQOL in patients resuming dialysis after graft loss, with poorer perceived HRQOL in women. This was apparent mainly for physical aspects. Gender inequity in self-perceived HRQOL with lower scores for women than men, particularly in physical aspects, has been reported in the general population [19,30], in patients with chronic kidney disease [31], and kidney transplanted patients [10,14,32]. Our study has extended the finding of gender differences in HRQOL to dialysis patients with functional renal graft loss. Despite the relative small number of female patients in our study, interaction analyses clearly indicate the importance of graft loss in women, with associations to reduced effect of kidney disease, physical function, cognitive function and general health. Losing the graft seems to influence HRQOL in females more negatively than in males. The explanation for this gender inequity is not readily apparent. Depression is known to be prevalent in the dialysis population [33,34], and previous studies have shown higher prevalence in females than in males [35]. Lopes et al [36] showed substantial lower HRQOL and more depressive symptoms in female patients, but the gender differences in HRQOL disappeared after adjustment for depression. We have previously shown depression to be prevalent also in the Norwegian dialysis population [21,34], but there was no gender difference [21]. As lower age is associated with depression in the dialysis population [21], adjustments for age were made in all regression analyses.

Patients with overt cognitive dysfunction were not included in the present study. To our surprise, we observed that older age was associated with better cognitive function in males only. Mild cognitive impairment is not uncommon and often underdiagnosed in dialysis patients [37]. As the item “cognitive function” was based on self-reported evaluation, the finding may reflect that younger patients have higher expectations to health than older. However, the KDQOL item “cognitive function” has been shown to be a poor determinant of neurocognitive performance in hemodialysis patients, with a low sensitivity and specificity [38].

If female patients with graft loss were more vulnerable than their male counterpart, transition to dialysis could lead to lower HRQOL. From the literature, the term response shift refers to adaptation to changing circumstances, even in patients with chronic diseases. This adaption process could be explained by changes in internal standards, values and conceptualization [39]. It has repeatedly been shown that patients with chronic diseases rate their health and quality of life better than what family members and physicians do when asked to assess the patients well-being [39,40]. Response shift has been described in kidney-pancreas transplanted patients, who retrospectively assessed how their HRQOL was before transplantation. The patients reported that HRQOL was lower than what was actually measured prior to the transplantation [41]. There is a paucity of data regarding response shift and gender differences. Our observation that women with graft loss resuming dialysis had lower HRQOL compared to men, could suggest a gender-specific response shift. This should be addressed in longitudinal studies.

A surprise observation in the present study was the high proportion of women resuming dialysis after functional graft loss. It is not known whether gender differences in graft- and patient survival, or inequity in waiting time for a second transplantation could explain this finding. Some studies show better patient and graft survival in women than men [42,43], but others find no gender inequities [44]. Thus, the reports of the influence of gender on graft and patient survival are inconsistent. A previous study from our group addressing the risk of death after renal transplantation did not reveal any gender differences [45]. As women on the waiting list for kidney transplantation are more likely to have panel reactive antibodies [46], finding an appropriate donor may prolong the waiting time. The number of patients in our study was low. Whether women have to wait longer for a second transplantation cannot be answered in this study, and should be addressed in a proper designed study with sufficient numbers of patients.

The strength of this study is that it is population-based, with patients from 10 different dialysis units with a catchment area of more than 2 million inhabitants, and represented almost 1/3 of the prevalent dialysis patients in Norway. All transplantations were done in one transplant center. The quality of the data was good as there were less than 2% missing data in the subscales of SF-36. Although only the healthiest of the dialysis patients could participate, the characteristics of the study population were similar to that of the general Norwegian dialysis population in respect to age, gender and cause of renal failure [23]. The major limitation is the restricted number of patients with renal graft loss in dialysis, but still many times higher than what have been reported in previous studies until recently [9-11]. Furthermore, most patients were Caucasian. As transplantation rate is high in Norway, also for a retransplantation, the time in dialysis is short compared to what has been observed in other populations. As time in dialysis and accessibility to transplantation may affect HRQOL, the generalizability of the results may be limited.

## Conclusion

Patients who had resumed dialysis due to functional graft loss did not have lower adjusted HRQOL than dialysis patients not previously transplanted after multiple adjustments. However, there is a significant interaction between gender and graft loss, and women in contrast to men experience lower HRQOL after graft loss. This novel and exciting observation of a gender disparity in the self-perceived HRQOL needs to be further explored.

## Abbreviations

RG -: Dialysis patients not previously kidney transplanted; RG +: Patients returning to dialysis after functional renal graft loss; HRQOL: Health related quality of life; SF-36: Medical Outcome Study 36-item Short Form Health Survey; KDQOL: Kidney disease quality of life; BMI: Body mass index; PCS: Physical component summary scale; MCS: Mental component summary scale; DOPPS: Dialysis Outcomes and Practice Patterns Study.

## Competing interests

The authors declare that they have no competing interests.

## Authors’ contributions

BW was involved in the statistical analyses, discussion and drafting of the manuscript. TBHØ collected data, contributed to the discussion and edited the manuscript. AVR contributed to the discussion of the data, and edited the manuscript. IO wrote the protocol, and supervised statistical analyses, the discussion and drafting of the manuscript. NvdL performed the statistical analyses, contributed to discussion and drafted the manuscript. All authors read and approved the final manuscript.

## Pre-publication history

The pre-publication history for this paper can be accessed here:

http://www.biomedcentral.com/1472-6874/14/34/prepub

## References

[B1] Mittal SK Ahern L Flaster E Maesaka JK Fishbane S Self-assessed physical and mental function of haemodialysis patients Nephrol Dial Transplant 2001 16 7 1387 1394 10.1093/ndt/16.7.1387 11427630

[B2] Yarlas AS White MK Yang M Saris-Baglama RN Bech PG Christensen T Measuring the health status burden in hemodialysis patients using the SF-36(R) health survey Qual Life Res 2011 20 3 383 389 10.1007/s11136-010-9764-8 20972630

[B3] Liem YS Bosch JL Arends LR Heijenbrok-Kal MH Hunink MG Quality of life assessed with the Medical Outcomes Study Short Form 36-Item Health Survey of patients on renal replacement therapy: a systematic review and meta-analysis Value Health 2007 10 5 390 397 10.1111/j.1524-4733.2007.00193.x 17888104

[B4] Fujisawa M Ichikawa Y Yoshiya K Isotani S Higuchi A Nagano S Arakawa S Hamami G Matsumoto O Kamidono S Assessment of health-related quality of life in renal transplant and hemodialysis patients using the SF-36 health survey Urology 2000 56 2 201 206 10.1016/S0090-4295(00)00623-3 10925078

[B5] US Transplant registry Internet 2013 http://srtr.transplant.hrsa.gov/annual_reports/2011/pdf/01_kidney_12.pdf

[B6] Reisaeter AV Foss A Hartmann A Leivestad T Midtvedt K The kidney transplantation program in Norway since Clin Transpl 2000 2011 111 118 22755407

[B7] Griva K Stygall J Ng JH Davenport A Harrison MJ Newman S Prospective changes in health-related quality of life and emotional outcomes in kidney transplantation over 6 years J Transplant 2011 2011 671571 21822474 10.1155/2011/671571 PMC3142681

[B8] Perl J Hasan O Bargman JM Jiang D Na Y Gill JS Jassal SV Impact of dialysis modality on survival after kidney transplant failure Clin J Am Soc Nephrol 2011 6 3 582 590 10.2215/CJN.06640810 21233457 PMC3082417

[B9] Johnson JP McCauley CR Copley JB The quality of life of hemodialysis and transplant patients Kidney Int 1982 22 3 286 291 10.1038/ki.1982.167 6757524

[B10] Jofre R Lopez-Gomez JM Moreno F Sanz-Guajardo D Valderrabano F Changes in quality of life after renal transplantation Am J Kidney Dis 1998 32 1 93 100 10.1053/ajkd.1998.v32.pm9669429 9669429

[B11] Maglakelidze N Pantsulaia T Tchokhonelidze I Managadze L Chkhotua A Assessment of health-related quality of life in renal transplant recipients and dialysis patients Transplant Proc 2011 43 1 376 379 10.1016/j.transproceed.2010.12.015 21335226

[B12] Griva K Davenport A Harrison M Newman SP The impact of treatment transitions between dialysis and transplantation on illness cognitions and quality of life - a prospective study Br J Health Psychol 2012 17 4 812 827 10.1111/j.2044-8287.2012.02076.x 22536819

[B13] Perl J Zhang J Gillespie B Wikstrom B Fort J Hasegawa T Fuller DS Pisoni RL Robinson BM Tentori F Reduced survival and quality of life following return to dialysis after transplant failure: the dialysis outcomes and practice patterns study Nephrol Dial Transplant 2012 27 12 4464 4472 10.1093/ndt/gfs386 23028105 PMC3616760

[B14] Rebollo P Ortega F Baltar JM Badia X Alvarez-Ude F Diaz-Corte C Naves M Navascues RA Urena A Alvarez-Grande J Health related quality of life (HRQOL) of kidney transplanted patients: variables that influence it Clin Transplant 2000 14 3 199 207 10.1034/j.1399-0012.2000.140304.x 10831077

[B15] Rosenberger J van Dijk JP Nagyova I Zezula I Geckova AM Roland R van den Heuvel WJ Groothoff JW Predictors of perceived health status in patients after kidney transplantation Transplantation 2006 81 9 1306 1310 10.1097/01.tp.0000209596.01164.c9 16699459

[B16] Silbiger S Neugarten J Gender and human chronic renal disease Gend Med 2008 5 Suppl A S3 S10 18395681 10.1016/j.genm.2008.03.002

[B17] System USRD 2011 Atlas of CKD & ESRD 2012 http://www.usrds.org/atlas12.aspx?zoom_highlight=atlas+2012

[B18] Registry E Chronic Kidney Disease-HEIDI WIKI 2012 https://webgate.ec.europa.eu/sanco/heidi/index.php/Heidi/Major_and_chronic_diseases/Chronic_kidney_disease

[B19] Audureau E Rican S Coste J Worsening trends and increasing disparities in health-related quality of life: evidence from two French population-based cross-sectional surveys, 1995-2003 Qual Life Res 2012 10.1007/s11136-012-0117-7 22298202

[B20] Molsted S Prescott L Heaf J Eidemak I Assessment and clinical aspects of health-related quality of life in dialysis patients and patients with chronic kidney disease Nephron Clin Pract 2007 106 1 c24 c33 10.1159/000101481 17409766

[B21] Osthus TB Dammen T Sandvik L Bruun CM Nordhus IH Os I Health-related quality of life and depression in dialysis patients: associations with current smoking Scand J Urol Nephrol 2010 44 1 46 55 10.3109/00365590903449324 20030569

[B22] Helsinkideclation Internet 2013 http://www.wma.net/en/30publications/10policies/b3/17c.pdf

[B23] Leivestad T Årsrapport fra Nefrologiregisteret 2010 2011 http://www.nephro.no/nnr/AARSMEL2010.pdf

[B24] Beddhu S Bruns FJ Saul M Seddon P Zeidel ML A simple comorbidity scale predicts clinical outcomes and costs in dialysis patients Am J Med 2000 108 8 609 613 10.1016/S0002-9343(00)00371-5 10856407

[B25] Grosso G Corona D Mistretta A Zerbo D Sinagra N Giaquinta A Tallarita T Ekser B Leonardi A Gula R Veroux P Veroux M Predictive value of the Charlson comorbidity index in kidney transplantation Transplant Proc 2012 44 7 1859 1863 10.1016/j.transproceed.2012.06.042 22974856

[B26] Hays RD Kallich JD Mapes DL Coons SJ Carter WB Development of the kidney disease quality of life (KDQOL) instrument Qual Life Res 1994 3 5 329 338 10.1007/BF00451725 7841967

[B27] Ware JE Jr Sherbourne CD The MOS 36-item short-form health survey (SF-36) I. Conceptual framework and item selection Med Care 1992 30 6 473 483 10.1097/00005650-199206000-00002 1593914

[B28] Ware JE Kosinski M Keller SD SF-36. Physical and Mental Health Summary Scales: A User’s Manual 1994 Boston: The Health Institute, New England Medical Center

[B29] Osthus TB Preljevic V Sandvik L Dammen T Os I Renal transplant acceptance status, health-related quality of life and depression in dialysis patients J Ren Care 2012 38 2 98 106 10.1111/j.1755-6686.2011.00254.x 21917125

[B30] Cherepanov D Palta M Fryback DG Robert SA Hays RD Kaplan RM Gender differences in multiple underlying dimensions of health-related quality of life are associated with sociodemographic and socioeconomic status Med Care 2011 49 11 1021 1030 10.1097/MLR.0b013e31822ebed9 21945974 PMC3687080

[B31] Mujais SK Story K Brouillette J Takano T Soroka S Franek C Mendelssohn D Finkelstein FO Health-related quality of life in CKD patients: correlates and evolution over time Clin J Am Soc Nephrol 2009 4 8 1293 1301 10.2215/CJN.05541008 19643926 PMC2723973

[B32] Liu H Feurer ID Dwyer K Speroff T Shaffer D Wright PC The effects of gender and age on health-related quality of life following kidney transplantation J Clin Nurs 2008 17 1 82 89 18088260 10.1111/j.1365-2702.2006.01745.x

[B33] Kimmel PL Psychosocial factors in dialysis patients Kidney Int 2001 59 4 1599 1613 10.1046/j.1523-1755.2001.0590041599.x 11260433

[B34] Preljevic VT Osthus TB Os I Sandvik L Opjordsmoen S Nordhus IH Dammen T Anxiety and depressive disorders in dialysis patients: association to health-related quality of life and mortality Gen Hosp Psychiatry 2013 35 6 619 624 10.1016/j.genhosppsych.2013.05.006 23896282

[B35] Lopes AA Albert JM Young EW Satayathum S Pisoni RL Andreucci VE Mapes DL Mason NA Fukuhara S Wikstrom B Saito A Port FK Screening for depression in hemodialysis patients: associations with diagnosis, treatment, and outcomes in the DOPPS Kidney Int 2004 66 5 2047 2053 10.1111/j.1523-1755.2004.00977.x 15496178

[B36] Lopes GB Matos CM Leite EB Martins MT Martins MS Silva LF Robinson BM Port FK James SA Lopes AA Depression as a potential explanation for gender differences in health-related quality of life among patients on maintenance hemodialysis Nephron Clin Pract 2010 115 1 c35 c40 10.1159/000286348 20173348

[B37] Kurella Tamura M Larive B Unruh ML Stokes JB Nissenson A Mehta RL Chertow GM Prevalence and correlates of cognitive impairment in hemodialysis patients: the Frequent Hemodialysis Network trials Clin J Am Soc Nephrol 2010 5 8 1429 1438 10.2215/CJN.01090210 20576825 PMC2924414

[B38] Sorensen EP Sarnak MJ Tighiouart H Scott T Giang LM Kirkpatrick B Lou K Weiner DE The kidney disease quality of life cognitive function subscale and cognitive performance in maintenance hemodialysis patients Am J Kidney Dis 2012 60 3 417 426 10.1053/j.ajkd.2011.12.029 22425261 PMC3547669

[B39] Sprangers MA Aaronson NK The role of health care providers and significant others in evaluating the quality of life of patients with chronic disease: a review J Clin Epidemiol 1992 45 7 743 760 10.1016/0895-4356(92)90052-O 1619454

[B40] Tsevat J Cook EF Green ML Matchar DB Dawson NV Broste SK Wu AW Phillips RS Oye RK Goldman L Health values of the seriously ill. SUPPORT Investigators Ann Intern Med 1995 122 7 514 520 10.7326/0003-4819-122-7-199504010-00007 7872587

[B41] Adang EM Kootstra G Engel GL van Hooff JP Merckelbach HL Do retrospective and prospective quality of life assessments differ for pancreas-kidney transplant recipients? Transpl Int 1998 11 1 11 15 10.1111/j.1432-2277.1998.tb00949.x 9503548

[B42] Adey DB Women and kidney transplantation Adv Chronic Kidney Dis 2013 20 5 427 432 10.1053/j.ackd.2013.06.008 23978549

[B43] Chen PD Tsai MK Lee CY Yang CY Hu RH Lee PH Lai HS Gender differences in renal transplant graft survival J Formos Med Assoc 2013 112 12 783 788 10.1016/j.jfma.2013.10.011 24246256

[B44] Vavallo A Lucarelli G Spilotros M Bettocchi C Palazzo S Selvaggi FP Battaglia M Ditonno P Impact of donor-recipient gender on kidney graft and patient survival: short- and long-term outcomes World J Urol 2013 in press 10.1007/s00345-013-1137-9 23907660

[B45] Oien CM Reisaeter AV Os I Jardine A Fellstrom B Holdaas H Gender-associated risk factors for cardiac end points and total mortality after renal transplantation: post hoc analysis of the ALERT study Clin Transplant 2006 20 3 374 382 10.1111/j.1399-0012.2006.00496.x 16824157

[B46] Hyun J Park KD Yoo Y Lee B Han BY Song EY Park MH Effects of different sensitization events on HLA alloimmunization in solid organ transplantation patients Transplant Proc 2012 44 1 222 225 10.1016/j.transproceed.2011.12.049 22310619

